# Human nutritional relevance and suggested nutritional guidelines for vitamin A5/X and provitamin A5/X

**DOI:** 10.1186/s12986-023-00750-3

**Published:** 2023-08-15

**Authors:** Torsten Bohn, Julian Hellman-Regen, Angel R. de Lera, Volker Böhm, Ralph Rühl

**Affiliations:** 1 Nutrition Research Group, Department of Precision Health, Luxembourg Institute and Health, 1 A-B, Rue Thomas Edison, 1445 Strassen, Luxembourg; 2 Department of Psychiatry, Charité-Campus Benjamin Franklin, Section Neurobiology, University Medicine Berlin, Berlin, Germany; 3 Departamento de Química Orgánica, Facultad de Química, CINBIO and IBIV, Universidade de Vigo, Campus As Lagoas-Marcosende, 36310 Vigo, Spain; 4 Institute of Nutritional Sciences, Friedrich Schiller University Jena, Jena, Germany; 5 CISCAREX UG, Transvaalstr. 27c, 13351 Berlin, Germany

**Keywords:** Nuclear receptors, Beta-carotene oxygenase, Recommended dietary allowance, Apo-carotenoids, Visual cycle

## Abstract

In the last century, vitamin A was identified that included the nutritional relevant vitamin A1 / provitamin A1, as well as the vitamin A2 pathway concept. Globally, nutritional guidelines have focused on vitamin A1 with simplified recommendations and calculations based solely on vitamin A. The vitamin A / provitamin A terminology described vitamin A with respect to acting as a precursor of 11-*cis*-retinal, the chromophore of the visual pigment, as well as retinoic acid(s), being ligand(s) of the nuclear hormone receptors retinoic acid receptors (RARs) α, β and γ. All-*trans*-retinoic acid was conclusively shown to be the endogenous RAR ligand, while the concept of its isomer 9-*cis*-retinoic acid, being “the” endogenous ligand of the retinoid-X receptors (RXRs), remained inconclusive. Recently, 9-*cis*-13,14-dihydroretinoic acid was conclusively reported as an endogenous RXR ligand, and a direct nutritional precursor was postulated in 2018 and further confirmed by Rühl, Krezel and de Lera in 2021. This was further termed vitamin A5/X / provitamin A5/X. In this review, a new vitamin A5/X / provitamin A5/X concept is conceptualized in parallel to the vitamin A(1) / provitamin A(1) concept for daily dietary intake and towards dietary guidelines, with a focus on the existing national and international regulations for the physiological and nutritional relevance of vitamin A5/X. The aim of this review is to summarize available evidence and to emphasize gaps of knowledge regarding vitamin A5/X, based on new and older studies and proposed future directions as well as to stimulate and propose adapted nutritional regulations.

**Supplementary Information:**

The online version contains supplementary material available at 10.1186/s12986-023-00750-3.

## The vitamin A concept

**Fig. 1:**
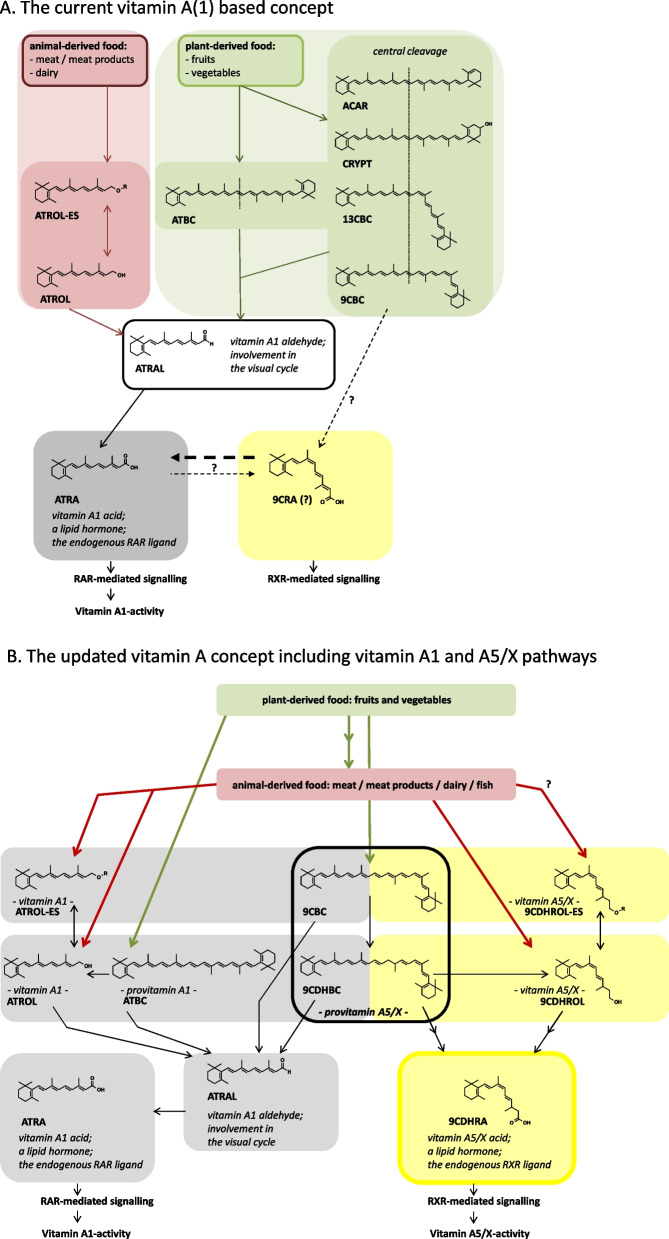
**A** The current vitamin A concept (the vitamin A1 concept): Summarized is the metabolic conversion starting from animal derived food products (at the left side with a reddish background), rich in vitamin A derivatives such as all-*trans*-retinyl esters (ATROL-ES) and all-*trans*-retinol (ATROL), which can be metabolized to the intermediate all-*trans*-retinal (ATRAL, a precursor of the visual pigment 11-*cis*-retinal, which performs important functions in the visual cycle) towards the active all-*trans*-retinoic acid (ATRA) as the bioactive metabolite of the vitamin A/vitamin A1 pathway. Besides (on the right side with a greenish background), the plant-derived food sources rich in provitamin A(1) are displayed with the double sided vitamin A(1)-precursor all-*trans*-β,β-carotene (ATBC) and additional carotenoids with a half-sided vitamin A(1) function such as all-*trans*-α,β-carotene (ACAR), all-*trans*-β,β-cryptoxanthin (CRYPT), 13-*cis*-β,β-carotene (13CBC) and 9-*cis*-β,β-carotene (9CBC), which are metabolized via the intermediate ATRAL to ATRA. In addition, in dashed lines, indicating a high uncertainty of this pathway, starting from 9CBC and ATRA to the potential as well as possibly to the endogenous RXR-agonist 9-*cis*-retinoic acid (9CRA). **B** The vitamin A5/X concept: Summarized is the metabolic pathway of the novel vitamin A5/X / provitamin A5/X pathway, starting from nutritional derived retinoids and carotenoids (on the left side with a grey blue background the bioactive metabolites of the vitamin A1 cluster pathway). On the right side (with a yellow background) the vitamin A5/X pathway with 9-*cis*-13,14-dihydroretinyl esters (9CDHROL-ES), as well as the direct nutritional precursor vitamin A5/X alcohol 9-*cis*-13,14-dihydroretinol (9CDHROL) and the provitamin A5/X carotenoids 9-*cis*-13,14-dihydro-β,β-carotene (9CDHBC) and 9-*cis*-β,β-carotene (9CBC), to vitamin A5/X acid, the lipid hormone, the endogenous RXR-ligand 9-*cis*-13,14-dihydroretinoic acid (9CDHRA) as the resulting bioactive ligand enabling RXR-mediated signalling

In the last century, the term vitamin has been used to describe and define essential micronutrients. As one of the first identified vitamins, all-*trans*-retinol and all-*trans*-β,β-carotene were named vitamin A and provitamin A, respectively. The initial importance of vitamin A in health was the prevention of ocular diseases mediated via the visual chromophore 11-*cis*-retinal [1–3]. Later on, vitamin A regained momentum because of its role in nuclear hormone signalling pathways mediated via all-*trans*-retinoic acid (ATRA) [4, 5]. The retinoic acid receptor (RAR)-mediated signalling was initially identified with ATRA (Fig. 1A), being a metabolite dependent on the nutritional intake of vitamin A / provitamin A [6]. These important investigations were awarded with various Nobel prizes. Frederick Hopkins received in 1929 the Nobel prize in Physiology and Medicine for the importance of growth-stimulating vitamins, with a focus on vitamin A. Paul Karrer won the Nobel prize in Chemistry for the structural identification of vitamin A in 1937 and George Wald followed with the Nobel prize in Physiology and Medicine awarded for demonstrating the importance of vitamin A in ocular health in 1967. In addition, in 2004 Pierre Chambon and Ron Evans received the Albert Lasker Award for Basic Medical Research for the identification of vitamin A / ligand activated nuclear hormone receptor mediated signalling, as reviewed previously [7, 8].

More recently, the detailed molecular signalling concept for specific disease prevention has been studied using “up to date” molecular-biological techniques [9–12]. Gene knock-out (KO)-mouse model studies confirmed the crucial retinoic acid receptor (RAR)-mediated vitamin concept [13, 14]. The primary application of this knowledge was the nutritional supplementation of human food or livestock feed with vitamin A / provitamin A to prevent vitamin A deficiency [15–18].

Further RAR-activators either of natural origin such as ATRA, as well as natural precursors, including retinol, retinyl-palmitate and retinal, were additionally included in pharmaceutical or cosmetic applications, while synthetic RAR-ligands such as Tigason™, Neotigason™, Adapalene™, Tazarotene™ and Trifarotene™ have been employed for systemic and/or local treatment of cancer or dermatological disorders [19–23]. β-Carotene is the most abundant provitamin A carotenoid in the human diet, and is used as a coloring agent, as an antioxidant and as a provitamin A carotenoid for human and animal food supplementation [24–26]. Provitamin A carotenoids are the major sources of dietary vitamin A in low- and middle-income countries and in populations following plant-based diets (including Western vegans and vegetarians) [24, 26].

The traditional focus on vitamin A research and nutritional recommendations has been put on the study of vitamin A1 derivatives. These included e.g. “all-*trans*”-retinol as vitamin A1 alcohol, “all-*trans*”-retinyl esters as the major vitamin A1 derivatives present in animal-derived food sources, the non-nutritional relevant “all-*trans*”-retinal as vitamin A1 aldehyde, the precursor of 11-*cis*-retinal, the active visual pigment in the human organism, as well as various provitamin A1 carotenoids present in vegetable-derived food sources (Fig. 1) [24, 26]. Surprisingly, the vitamin A2 cluster (namely 3,4-dehydroretinol) has been much less investigated, likely because of a lower physiological and nutritional relevance for humans [27–31]. By contrast, in birds and fish, vitamin A2 seems to be of major important physiological and nutritional relevance [28, 32]. Vitamin A3 and A4 are retinal analogues serving as visual pigments for arthropods or crustaceans, solely of relevance for non-mammalian species [33]. The current vitamin terminology was exclusively established with a focus on human relevance [34], which poses the question of whether the vitamin A terminology is appropriate, as vitamin A3 and A4 have apparently no human relevance and one can question if they should be thus termed vitamins.

Recently, a new vitamin A cluster was identified, and as the terms vitamin A3 and A4 were already used, suggested and termed vitamin A5 / provitamin A5 [35]. The term “vitamin A5” was used and based on subordination in the existing vitamin A cluster, due to a familiar retinoid / carotenoid background and slightly overlapping metabolism and mechanisms of signalling. Unfortunately, the subordination of vitamin A5 only under the vitamin A category would partly result in non-conclusive and uncertain nutritional regulatory recommendations, overlapping with existing vitamin A / vitamin A1-guidelines [34, 36, 37]. The currently used vitamin A concept, including dietary recommendations and fortification regulations, do exclusively rely on the vitamin A1 pathway. For practical reasons, its name was replaced by the simplified term vitamin A. Therefore, alternatively, because of functioning as a nutritional precursor of the RXR-activation pathway, the letter “X” was chosen, suggesting a new unique vitamin category, and named “vitamin X”.

As reviewed earlier, alternative endogenous and physiologically-relevant RXR-ligands and activators have been described [38]. The two main alternative candidates 9-*cis*-retinoic acid (9CRA) and the non-esterified/free fatty acid docosahexaenoic acid (DHA), failed to demonstrate to act as candidates for RXR-ligands at physiological level. This would comprise the criteria food intake, uptake, metabolism, being present in sufficient concentrations for receptor activation at the cellular level and eliciting nuclear hormone receptor (NHR)-mediated signalling via the RXR-receptor [38]. Based on various gaps and an overall non-conclusive concept, their relevant function as “a” or “the” physiological RXR-ligand is highly unlikely for RXR-mediated signalling at levels as occurring in food items. However, their relevance cannot be fully excluded. The vitamin A5/X / provitamin A5/X concept proposes novel identified physiologically- and nutritionally-occurring precursors of the recently identified lipid hormone, 9-*cis*-13,14-dihydroretinoic acid (9CDHRA), conclusively identified currently as the only physiologically relevant RXR-ligand (Fig. 1B) [39–43].

## The current vitamin A national and international recommendations

At present, several dietary intake recommendations for vitamin A exist. These are generally expressed for pre-formed vitamin A as retinol-equivalents (RE) or as retinol activity equivalents (RAE) (Table 1 and Additional file 1: Table 1). Among the internationally most important ones are the USDA-DRI (dietary reference intakes) and for Europe EFSA’s (European Food Safety Authority) DRVs (dietary reference values), both of which are umbrella terms with a set of recommendations. The EFSA suggested population reference intakes (PRI) as part of the DRVs, which are recommendations of nutrients for the general and healthy population, according to gender and age groups, including vitamin A but not carotenoids. These PRIs are intended to cover the needs of a nutrient of approx. 97.5% of persons within a population (i.e. for a specific age and gender group), and are derived from the estimated average requirements (AR) plus adding 2 times the standard deviation of the population’s need. The same is true for the USDA derived RDA (recommended dietary allowance) which is obtained from the EAR (estimated average requirement).

**Table 1:** Intake recommendations for vitamin A and provitamin A / β-carotene (**A**) Selected intake recommendations for vitamin A in vitamin A equivalents (VAE), all individual values from international and national organizations are presented in Additional file 1: Table 1. (**B**) Selected intake recommendations for β-carotene

	National RDI’s or similar for vitamin A (µg RE/d or µg RAE/d for DACH)	National UL or similar for vitamin A (µg RE/d)
(*A*)		
*Range*		
Adult man	600–900	3000
Adult woman	500–750	3000
*Range*		
Pregnant woman	700–800	3000
*Range*		
Lactating woman	850–1300	3000
*Range*		
Children	300–950	600–2800

Organization	National RDI or similar for β-carotene (mg/d)	National UL or similar for β-carotene (mg/d)
(*B*)		
EFSA^5^	N/A	15
UK-EVM-GL & SUL^6^	N/A	7
DGE^7^	2	N/A
DGE-vegetarians^7^	8	N/A

All ranges are based on EFSA^1^, USDA^2^, WHO-FAO^3^ and DACH^4^ recommendations listed in Additional file 1: Table 1

References: ^1^ EFSA: DRV-PRI and UL [145]; ^2^ DRI-RDA and UL [146]; ^3^ WHO/FAO: Safe recommended intake [34, 45], ^4^ DACH Recommended Dietary Intakes [44]; ^5^ EFSA—safe intake (including smokers from additives & supplements) [46]; ^6^ UK-Safe upper levels for vitamins and minerals as proposed by the expert group for vitamins and minerals [147]; ^7^ DGE-RDIs [42]

DACH, German, Austrian and Swiss Recommended Dietary Intakes; DGE, German Nutrition Society; DRI, dietary reference intakes; DRV, dietary reference values; EFSA, European Food Safety Authority; GL, guidance level; PRI, population reference intakes; RDA, recommended dietary allowance; RDI, recommended daily intake; RAE, retinol activity equivalents; RE, retinol equivalents; SUL, safe upper level; UL, tolerable upper intake level; USDA, US Department of Agriculture; WHO-FAO, World Health Organization/Food and Agriculture Organization

Besides being different between national institutions, these intake recommendations also differ according to gender and age groups (Table 1 and Additional file 1: Table 1). For preformed vitamin A, EFSA’s PRI values vary between 250 µg/d RE for infants and 1300 µg/d for lactating women. The USDA-RDA values are very similar; though tend to be slightly higher. The German/Austrian/Swiss recommendations (RAE) are similar, up to 1300 µg/d for lactating women. Lowest recommendations generally exist for infants, and highest for lactating followed by pregnant women. Special recommendations for elderly are generally not defined.

For carotenoids, including β-carotene, there are no umbrella recommendations such as DRI or DRV. For provitamin A in the form solely of all-*trans*-β,β-carotene, an intake of 6 mg/d was suggested for men older than 19 years and 4.8 mg/d for women older than 19 years [44]. These take into consideration the equivalence of 1 mg of retinol with 6 mg of β-carotene and 12 mg of other provitamin A carotenoids.

Based on adverse effects of various supplementation trials in smokers, a safe intake of < 15 mg/d from supplements and food additives was recommended by the EFSA. These considered both smokers and the general population [45, 46]. This was established after an increased cancer incidence was found in two major supplementation trials with doses of 20–30 mg/d of β-carotene over several years in human smokers [47, 48]. These studies demonstrated the cancerogenic effects of smoking and the risk of supplementation in heavy smokers. Consequently, it became clear that β-carotene is not an anti-cancer protection dietary supplement reverting and preventing detrimental effects of smoking. Ferret based animal models showed reduced ATRA-levels in the lung of smoke exposed ferrets supplemented orally with high doses of β-carotene in corn oil [49, 50], likely due to a strong bio-feedback mechanism [51].

Even high supplemented amounts of β-carotene have been safely given for a semi-therapeutic application of up to 240 mg/person/d during 6 months for a potential treatment of cystic fibrosis [52]. Some clinics such as the US Mayo clinic recommend 30–300 mg/d for the prevention of negative reactions to sun in subjects with erythropoietic protoporphyria [53, 54]. The only side-effect noted for high carotenoid intake (above ca. 30 mg/d) was carotenodermia, i.e. a reversible orange discoloration of the skin [55]. This discoloration is partly even seen positive, as a healthy sun tanning coloration of the skin by large parts of the population.

In general, vitamin A can be sub-divided into two nutritionally-relevant precursor pathways; preformed vitamin A (retinol and retinyl esters) and provitamin A (all-*trans*-β,β-carotene, previously simplified as all-*trans*-β-carotene and even more simply as β-carotene, and alternative provitamin A carotenoids such as α-carotene, 13-*cis*-/9-*cis*-β,β-carotene and β-cryptoxanthin) (Fig. 1A) [56]. Based on this vitamin A concept, EFSA provides specific recommendations for reaching vitamin A PRI levels for retinol, i.e. 0.65–0.75 mg/d for normal adults (Table 1 and Additional file 1: Table 1), and based on conversion ratios for provitamin A carotenoids. Conversion rates vary due to matrix interactions and host factors, thus various equivalence doses may be calculated [25]. Due to half the equivalence of β-carotene from a bioavailable source, such as for β-carotene in oil of 1.3–1.5 mg/d for this provitamin A carotenoid [57], rather low doses would cover vitamin A needs. Due to lower bioavailability from other food matrices, and equivalences of up to 28:1 for β-carotene [58], equivalent doses of 18.2–21 mg/d would be required. Based on an often-employed equivalence of 1:12 proposed by the US-Institute of Medicine (IOM), for pre-formed vitamin A vs. β-carotene, a dose of 10.8 mg/d would be needed for adult males to meet the USDA requirements if all vitamin A would be derived from this provitamin A carotenoid. Again, these are average values for the general population, with a large uncertainty range, and should be considered with care at the individual level.

**Fig. 2:**
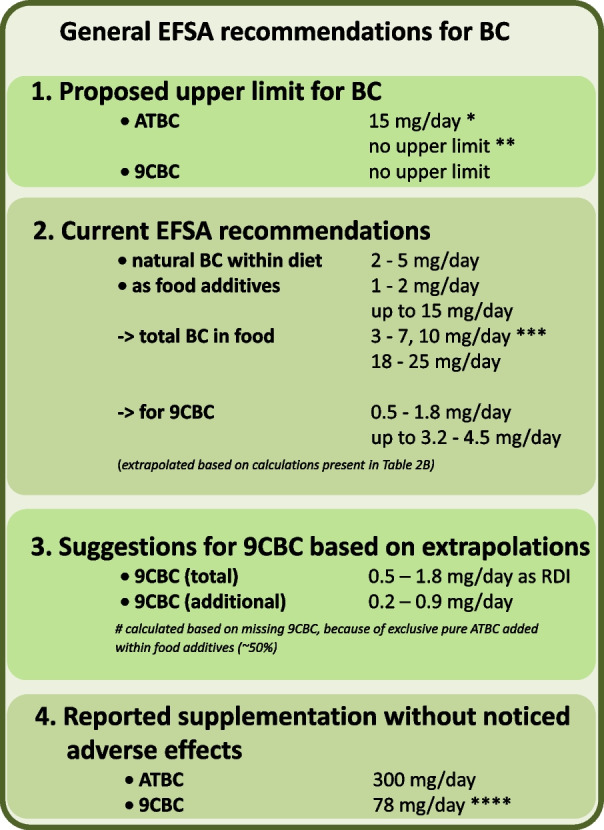
Current EFSA recommendations for β-carotene (BC) with a focus on all-*trans*-β,β-carotene (ATBC) and our suggestions for 9-*cis*-β,β-carotene (9CBC). # based on our own calculations/percentile amounts based on calculations originating from Table 2b, * relevant only when the individual is a tobacco smoker (no proposed upper limit for general population) and only a recommendations for people who are tobacco smoking in parallel to taking high-dose BC supplementation/consumption, ** relevant, when the individual is not a smoker; *** based on 10 mg/d as upper value due to EFSA suggestion [43, 70]; **** volunteers with single treatment, Stahl et al. [136] (Table 3)

When comparing these recommendations with intake data, the latter is typically lower. The average daily intakes of β-carotene for the general population were estimated as: (a) β-carotene from food additives as 1–2 mg/d (we assume pure all-*trans*-β,β-carotene is meant), (b) β-carotene from natural food sources as 2–5 mg/d and, thus resulting in a total β-carotene daily intake of 3–7 mg and up to 10 mg/d, taking into account seasonal variations [59]. In a recent publication summarizing various large-scale observational studies reporting carotenoid intake, an average intake of 4.8 mg/d for β-carotene was reported [26], as summarized in Fig. 2.

When comparing the β-carotene recommendations of 7 mg/d for a safe intake with an estimated real intake of approx. 5–10 mg/d, the recommendation “window” for supplementation remains rather small, at least for smokers. This is based on calculations with additional dietary supplements to the maximum of 15 mg (from supplements and food additives as proposed by EFSA) and is further based on the negative effects of 20 mg/d observed in smokers in the ATBC study. Alternatively, considering a low average intake of fruits and vegetables and being a non-smoker, then the “window” for supplementation is wide open.

## Problems with the current vitamin A recommendation

In chemical terms, the vitamin A1 umbrella terminology includes “all-*trans*-β”-retinol / “all-*trans*-β”-retinyl esters, “all-*trans*-β,β-carotene” and further likely nutritional minor important provitamin A1 precursor carotenoids, however only the term “vitamin A” is currently explicitly mentioned. There is an assumption that the “all-*trans”*-configuration is always the point of reference [34, 36, 37].

The concept of vitamin A2 [29–31] was originally identified in the nineteenth century alongside vitamin A1 [16–18, 60–62], but has received little to no scientific interest. Neither the nutritional relevance nor the molecular signalling mechanisms were further examined. Due to this, we speculate that omitting the vitamin A1 and A2 concept as sub-categories of vitamin A was suggested and executed because of a previously and currently non-felt importance. Therefore, no further specific A1 and A2 nutritional guidance and individual recommendations were given and executed [34, 36, 45].

The previously used nutritional RXR-ligand precursor concept was in the first instance associated with the general vitamin A / vitamin A1 category. This postulated a simple isomerization of the active ligand all*-trans*-retinoic acid (ATRA) to 9-*cis*-retinoic acid (9CRA) [40, 63–67]. In reverse, an isomerization of the relatively unstable 9CRA back to ATRA is more likely [68], as shown in Fig. 1A. 9CRA might further bind and activate the RXR [63, 64] to enable further RXR-mediated signalling. While this concept was published in highly ranked prestigious journals and was always well cited with > 2000 citations until August 2022, various experts in retinoid lipidomics could not conclusively confirm the physiological presence of 9CRA in the human organism, as reviewed recently [40]. Additionally, a substantial nutritional-metabolic conversion and activation cascade starting from all-*trans*-retinol / all-*trans*-β,β-carotene towards physiologically- and nutritionally-relevant levels of “the endogenous RXR ligand” 9CRA was neither shown nor conclusively confirmed [67–70]. These concepts were mainly based on identification techniques available in the 80´ies for which the 9CRA detection capacity was limited.

Furthermore, a study using an *ex vivo* human intestinal mucosa model “identified”, via simple LC-UV co-elution analytical determination, that 9CBC can be cleaved to “9CRA” [70]. The analytical system used a methodology with strong shifting retention times for ATRA, ranging between 5.8 and 6.8 min. and for 9CRA the range was between 5.9 and 6.5 min. (calculated based on Fig. 1A, [70]). As previously discussed in detail [40], this study used outdated methodologies that lacked robust parallel identification techniques for a conclusive identification of 9CRA.

In summary, based on the methodological issues surrounding the identification of 9CRA and its direct upstream nutritional precursors, a novel alternative 9CDHRA / vitamin A5/X concept was recently suggested [40] and later even confirmed [35]. This is important because a conclusive and irrefutable identification of 9CDHRA has physiological and nutritional implications for general vitamin A science. The current vitamin A concept and this current recommendation status should now be optimized and better explained, by sub-categorizing this current vitamin A concept into specific vitamin A1 as well as vitamin A5/X concepts.

Vitamin A2 is currently not sub-categorized and nutritional recommendations are not suggested. In addition, no further deeper investigation about food sources and mechanisms of action are clearly known, as there is a large overlap between the vitamin A1 and vitamin A2 pathways regarding RAR-ligands precursors [40] and a likely non-relevant function of vitamin A2 as a human chromophore. Based on food intake of vitamin A2 and impact on vitamin A2-induced signalling in the human body, we believe that there is no strong need for further targeted investigations into this direction.

On the other hand, vitamin A5/X seems to be a highly important new vitamin A concept. Vitamin A5/X is a novel precursor concept for the physiologically- and nutritionally-relevant RXR-ligand, 9CDHRA, for a further highly important initiation, maintenance and a general regulation of RXR-mediated signalling in the human organism [7–9, 39].

## The new player and the new concept of a new vitamin; vitamin A5/X / provitamin A5/X

The major importance of vitamin A5/X / provitamin A5/X is to act as a nutritional precursor of the endogenous RXR-ligand 9CDHRA. The precursor concept is similar to the well-established role of retinol and retinyl esters acting as vitamin A1 for further bioactive vitamin A1 derivatives, such as retinal and ATRA (Fig. 1A). In the case of vitamin A5/X, the alcohol form 9-*cis*-13,14-dihydroretinol (9CDHROL) and the ester form both act as precursors for 9-*cis*-13,14-dihydroretinoic acid (9CDHRA) (Fig. 1B) [35]. Similar to vitamin A1 precursors, 9CDHROL precursors are also likely to originate mainly from dietary ingestion of foods from animal origin such as meat, fish and dairy products (Fig. 1A) [35]. No in-depth analysis of these food sources was carried out to date but are the topic of ongoing examination.

In parallel, for vitamin A5/X, similar carotenoid precursors, namely β-carotene and alternative provitamin A1 precursors exist. These were identified as the provitamin A5/X precursor 9-*cis*-β,β-carotene (9CBC) with its proximate intermediate provitamin A5/X precursor 9-*cis*-13,14-dihydro-β,β-carotene (9CDHBC) [35]. 9CBC is present in the human organism in serum, in various organs and in human breast milk (Table 2A). It is also found in different isomer ratios with all-*trans-*β,β-carotene (ATBC) in different concentrations in a variety of vegetable-derived food sources (Table 2B). Here, a special nutritional focus seems to be placed on leafy and root vegetables, which are rich in this compound.

**Table 2:** (**A**) Concentrations of ATBC and 9CBC in human serum, breast milk and further analyzed organs. (**B**) Selected examples of ATBC and 9CBC as present in fruits and vegetables

(*A*)	ATBC (nM)		9CBC (nM)		% of 9CBC^a^
Serum	131/275/703^b^		UDL^b^		–
	420 (120–890)^c^		0–4 ^c,d^		0–1^c,d^
Liver	1400/1600/7300^b^		400/600/2100^b^		22/27/22
	3020 (160–8620)^c^		1162^c,d^		28 ^c,d^
Kidney	550 (80–2031)^c^		177^c,d^		24 ^c,d^
Adrenals	1800/2000/4400^b^		100/100/300^b^		5/5/6
	5600 (680–31,830)^c^		988^c,d^		15 ^c,d^
Testes	2680 (750–4770)^c^		670^c,d^		29^c,d^
Prostate	480 ± 0.06^e^		380 ± 0.06^e^		~ 44
Breast milk	916^f^		24^f^		~ 3

(*B*)	Dry weight in µg/g		Fresh wet weight in µg/g		% 9CBC of total BC fresh weight^m^
	ATBC	9CBC	ATBC	9CBC	
*Fruits*					
Peach^o^		2.2	0.3	12	
Apricots^n^	16.0^g^	4.4^g^	2.0	0.6	23
Papaya^n^	10.6^h^	7.0	1.9	1.3	41
*Vegetables/root vegetables*					
Pumpkin^n^	9.7^i^	2.7	0.6	0.2	28
Sweet potato^p^			38.2	1.5	4
Tomato^o^			71.0	4.8	6
Carrots^n^	1030^j^	57.1^j^	117	6.5	5
*Leafy vegetables*					
Lettuce^n^	104^k^	41.0^k^	5.0	2.0	29
Broccoli^o^			29.2	5.0	15
Kale^q^			59.4	14.1	19
Spinach^o^			311.9	38.6	11
**Calculated total average for fruits and vegetable**					**18**
*Algae with no direct food relevance*					
Dunaliella salina^n^	38,500	37,800	^l^	^l^	50

(A) (^a^) The percentage of ATBC and 9CBC of total BC; (^b^) Stahl et al. [148]; (^c^) Stahl et al. [149]; with (^d^) approx. calculations of 9CBC concentrations based on the displayed figures; (^e^) Clinton et al. [150]; (^f^) Johnson et al. [151]; UDL - under detection limit. (B) Individual dry weight listed as (^g^) 12.6% (^n^), (^h^) 18.3% (^n^), (^i^) 6.2% (^n^); (^j^) 11.3% (^n^), (^k^) 4.8% (^n^), (^l^) not calculated because the wet algae and thereby the weight of these algae are not of nutritional relevance. (^m^) total BC = ATBC + 9CBC; (^n^) Ben-Amotz and Fishler [152], (^o^) Khoo et al. [153], (^p^) Berni et al. [154], (^q^) Murador et al. [155]

## The invisible provitamin, 9-*cis*-β,β-carotene: from a “substitute” to the “most valuable player” in the vitamin A-team

Vitamin A5/X comprises 9CDHROL plus additional potential 9CDHROL-esters. Currently, only the vitamin A5/X alcohol (9CDHROL) was identified in beef liver (8 ng/g) [35], representing a human food source. Our group is working to further determine the vitamin A5/X content in additional animal derived foods, including different meat sources, meat products, marine-/fresh-water fish as well as different sources of milk and dairy products.

Additionally, 9CBC acts as a double-edged sword being a unique precursor for enabling further vitamin A1- and A5/X-mediated signalling, functioning as a precursor for the RAR ligand ATRA on one side and as a precursor for the RXR ligand 9CDHRA on the other side. Based on its provitamin A5/X precursor, 9CBC is claimed to be “the” nutritionally relevant provitamin. The contents of 9CBC in human blood serum, tissues and human breast milk, as well as in various food items, are shown in Tables 2A and B. Both tables also present the contents of ATBC. Using these data, the relative portion of 9CBC was calculated in relation to ATBC. Human blood serum only contained very low amounts (0–1% expressed as a percentage of total β-carotene) of 9CBC, while in organs such as liver, kidney and testes high relative proportions (22–28%) of 9CBC were found and lower amounts of approx. 3% 9CBC in breast milk (Table 2A).

This indicates also that 9CBC is an almost “invisible” derivative, as blood serum/plasma levels are very low compared to organ levels and seem to reflect just a minor proportion of the nutritional intake of 9CBC. We speculate that after ingestion of food high in 9CBC there is a quick rise of 9CBC in plasma or serum levels and further 9CBC is either stored in tissues and/or quickly metabolized to 9-*cis*-13,14-dihydro-retinoids in the human organism. Due to this problem, other options for detecting a healthy vitamin A5/X / provitamin A5/X dietary intake and status must be chosen, such as alternatively monitoring the active vitamin A5/X derivative, 9CDHRA, in the easily accessible serum/plasma compartment or choosing harder accessible compartments of the human organism such as white blood cells or organ biopsies for direct provitamin A5/X analysis.

Regarding dietary intake, fruits, papaya (41%) and apricots (23%) showed the highest relative portions of 9CBC (expressed as a fraction of total β-carotene) in fruits, while being low in total concentration of 1.9–2.0 µg/g of wet weight (Table 2A). Lettuce (29%), pumpkin (28%) and kale (19%) were the vegetables with the highest relative portions of 9CBC. Highest concentrations of 9CBC were found in leafy vegetables including spinach (312 µg/g) and kale (59 µg/g), as well as root vegetables such as carrots (117 µg/g), while levels in fruits were relative low, for instance in papaya (2 µg/g) and apricots (2 µg/g). The selected fruits and vegetables shown in Table 2B were used to calculate a mean content of 9CBC of 18% used for further calculations in Table 2B and Fig. 2/7.

One major question remains, namely at which levels does this specific all-*trans*- to 9-*cis*-isomerization takes place? The first step is a targeted synthesis of 9-*cis*-carotenoids in plants/algae. In *Dunaliella salina,* rich in 9CBC, specific β-carotene isomerases [71] and breeding conditions with specific environmental conditions/stimuli, such as illumination with red light and avoidance of energy rich purple/blue light illumination [72], strongly promote 9-*cis*-isomerization and accumulation. However, at which specific stage of algae 9CBC-synthesis this 9-*cis*-isomerization occurs is still unclear. If similar mechanisms occur in plants, especially in plants that constitute food for humans, is not clear. In general, these environmental stimuli such as light irradiation and physiologically-relevant thermal interference might also be of relevance in plants under certain still not observed and investigated conditions. Recently, an enzyme (DWARF27) was identified [73] and associated with a specific 9-*cis*-isomerization of all*-trans*-β-carotene to 9-*cis*-β-carotene [74]. Again, its relevance in plants with food-relevance for humans remains unclear.

As a second option, a specific isomerization of all-*trans-*carotenoids to 9-*cis*-carotenoids in mammals has, to the best of our knowledge based on available literature and our own experiments, not been observed.

In our view, the most important mechanism of human relevance is an unspecific isomerization of ATBC to 9CBC during food processing such as cooking (> 100 °C) [75]. It was shown that 9CBC increased in total amounts and expressed as a fraction [76] and it was shown to be 3–4 higher after cooking, depending among other on time and temperature [77].

Further food supplementation trials were carried out with preparations rich in 9CBC and were mainly based on carotenoids derived from algae (Table 3), ranging from ~ 10 to 78 mg 9CBC/d. In all these supplementation trials, no adverse effects were associated with the supplementation of β-carotene.

**Table 3:** Supplementation trials with preparations high in 9CBC

Author	Description/dose	Isomer ratio and 9CBC amount	Purpose of study and beneficial activity outcome
Stahl et al. [156]	5.6 µmol BC/kg bw as a **single dose** of Betatene™; ~ 70 kg bw and ~ 5.6 µmol BC (~ 210 mg BC)	54% ATBC: 37% 9CBC **- > ~ 77.7 mg 9CBC/day**	Absorption/accumulation study
Johnson et al. [151]	**Single treatment** with dried algae powder	64 mg ATBC (48%): 69 mg 9CBC (52%) **- > ~ 69 mg 9CBC/day**	Carotenoid nutri-kinetic study
Shaish et al. [157]	Natural derived extract rich in BC with 60 mg/BC day for 6 weeks **(42 days)**	~ 50% ATBC: 50% 9CBC **- > ~ 30 mg 9CBC/day**	Increase of HDL-cholesterol in fibrate treated patients
Rotenstreich et al. [158]	Treatment for **90 days** with capsules containing 300 mg 9CBC-rich dried algae containing ~ 20 mg BC	~ 50% ATBC: 50% 9CBC **- > ~ 10 mg 9CBC/day**	Increased retinal functions in patients with Retinitis Pigmentosa
Meshi et al. [159]	1200 mg BC rich powder with ~ 20 mg BC content for 3 months **(~ 90 days)**	~ 50% ATBC: 50% 9CBC **- > ~ 10 mg 9CBC/day**	Improvement and partly recovery of vision loss after ocular quintine toxicity

## Current governmental regulations focusing specifically on provitamin A1, β-carotene and biological relevant extracts rich in β-carotene for human nutrition

Based on the general regulation and the current applied terminology, the term provitamin A describes derivatives that can be transformed via metabolic activation to vitamin A [34]. This provitamin A1 term comprises all-*trans*-β,β-carotene and its isomers, such as 13-*cis*-β,β-carotene, 9-*cis*-β,β-carotene and minor potential endogenously occurring mono- or even di-*cis*-isomers. Besides, α-carotene (correctly all-*trans*-α,β-carotene) and β-cryptoxanthin are also included, while no geometric isomers of nutritional relevance have been described [34]. 9CBC is thereby a known provitamin A / provitamin A1 carotenoid with a low average presence in human food ingredients when expressed as the fraction of total β-carotene [35, 45, 78]. This may mean in consequence that besides ATBC, also 9CBC can be considered as a safe food compound with provitamin A activity as described in EFSA regulations about mixed carotenes [45]. β-Carotene is present on the food ingredient market in various forms of applications such as E160a in the sub-categories E160a(i, ii, iii, iv), as described in codex-alimentarius [36]. Surprisingly, the EFSA nomenclature was different, indicating mixed carotenes as E160a(i) and synthetic β-carotene as E160a(ii) [45]. In a correct summary based on the codex alimentarius guidelines; E160a(i) describes chemically synthesized all-*trans*-β,β-carotene, E160a(ii) describes vegetable-derived β-carotene, E160a(iii) describes *Blakeslea trispora* (a fungi)-derived β-carotene and E160a(iv) describes “algal-derived β-carotene” [36]. This nomenclature was later corrected and implemented and amended by the ESFA [59]. This E160a(iv) definition was recently altered in the codex alimentarius to “β-carotene-rich extracts from *Dunaliella salina*” [79]. In summary, these non-synthetic carotenoids here focused on algae origin (*Dunaliella salina)* are safe and legally approved for food supplementation within specific frames and guidelines in the EU [80] plus further annual amendments [81], as well as by the USA-FDA (GRAS000351) for *Dunaliella (bardawil) salina* [82].

These mixed carotenoids are usually used as general provitamin A as well as mainly yellowish/orange food colorants. This suggests that 9CBC is already present at low amounts as a food ingredient in the EU and US and is approved by various national and international governmental authorities. E160a(iv) is currently only used in minor amounts as a food ingredient and colorant due to a much higher price compared to E160a(i).

In summary, 9CBC is present in low percentage amounts in natural foods as well as in food ingredients and as such is a well-known, safe and approved and governmental licensed food ingredient.

## Importance of RXR-mediated signalling in the human organism

**Fig. 3:**
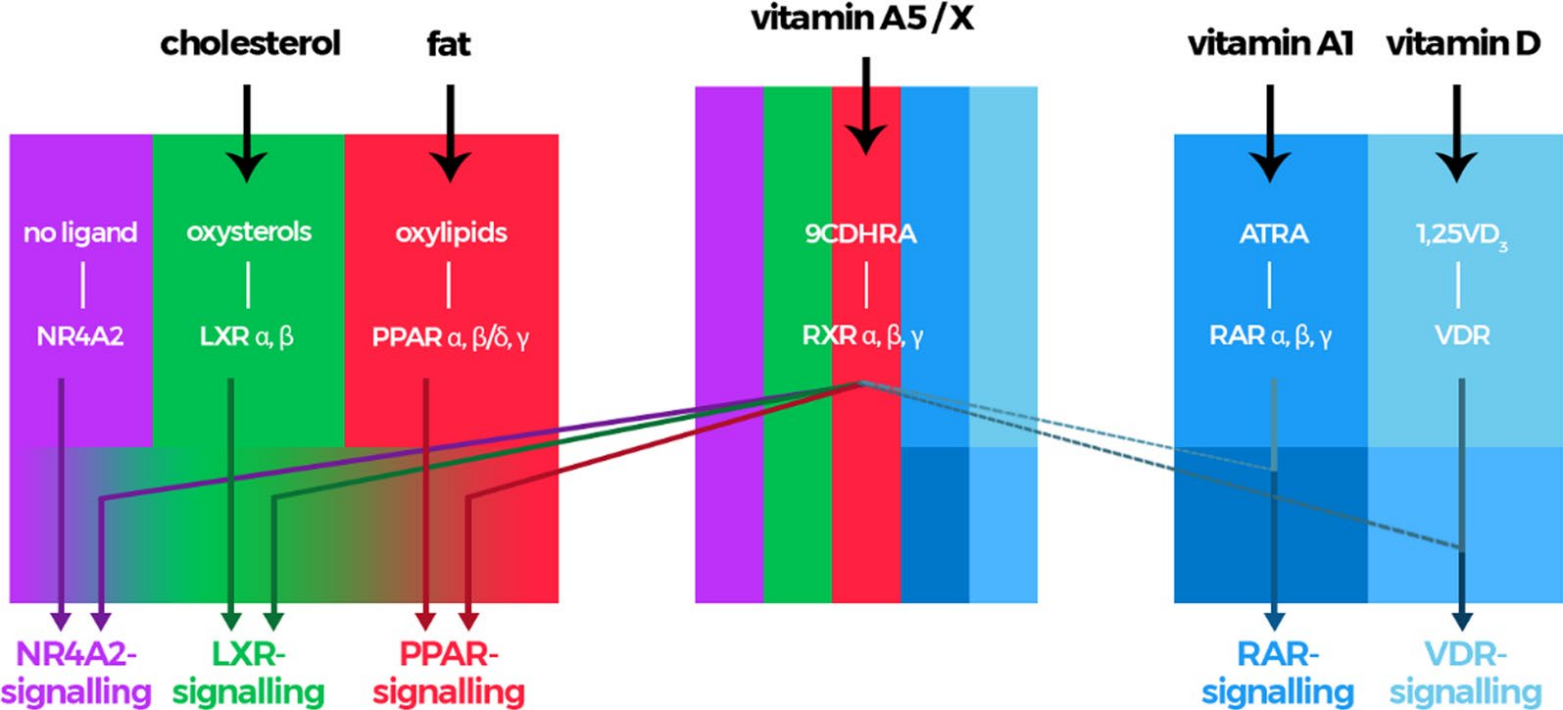
Nuclear hormone receptor mediated signalling with ligand activation by RXR-heterodimers with permissive partners including nuclear receptor subfamily 4 group A member 2 (NR4A2/Nurr1), peroxisome proliferator-activated receptor (PPARs) and liver X receptor (LXRs) and non-permissive partners such as vitamin D receptor (VDR) and retinoic acid receptors (RARs). Abbreviations; 9-*cis*-13,14-dihydroretinoic acid (9CDHRA), all-*trans*-retinoic acid (ATRA) and 1,25-dihydroxy-vitamin D_3_ (1,25VD_3_)

**Fig. 4:**
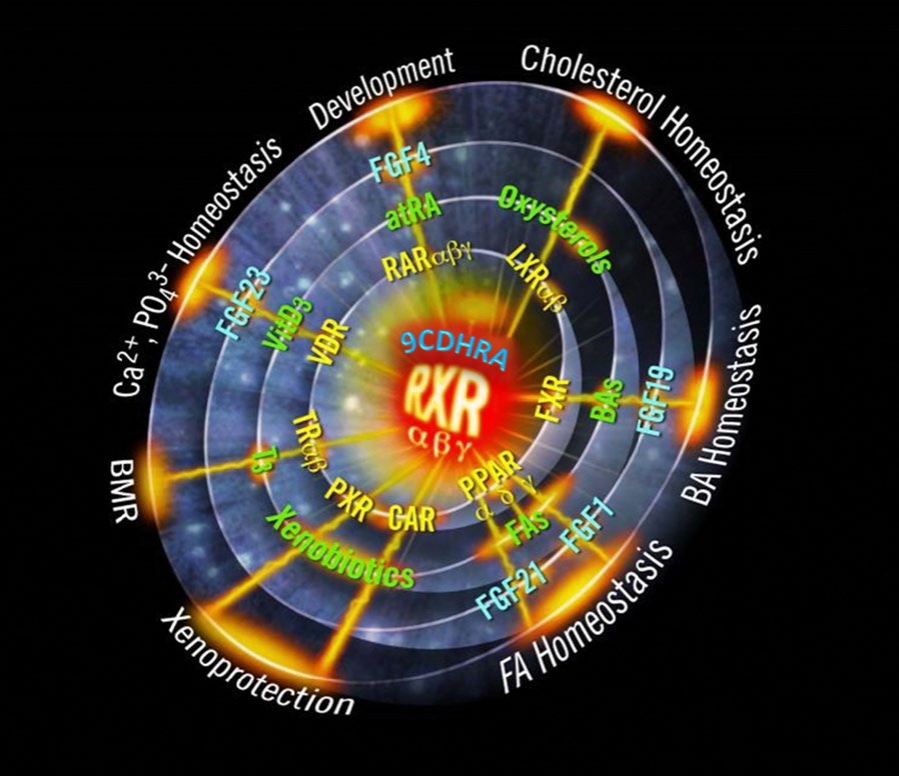
Summarized relevance of various nuclear hormone receptor mediated signalling pathways ranging from retinoic acid receptor (RARα,β,γ), liver X receptor (LXRα,β), farnesoid receptor (FXR), pregnane X receptor (PXR), constitutive androstane receptor (CAR), thyroid receptor (TRα,β), vitamin D receptor (VDR) and peroxisome proliferator-activated receptor (PPAR) interacting with the RXRs α,β,γ to mediated described physiological response pathways in the mammalian organism. Based on Evans and Mangelsdorf [9], with adaptations

The RXR is the crucial binding partner for other heterodimer-partners of the nuclear hormone receptor group [9, 10, 83, 84]. There is a large variety of these nuclear hormone receptors (NHR), while we mainly focus here on the major known ones with a direct “health” and “food”-application potential, i.e. the retinoic acid receptors (RARs), peroxisome proliferator-activated receptors (PPARs), liver X receptors (LXRs), farnesoid X receptors (FXR), vitamin D receptor (VDR) and nuclear receptor subfamily 4 group A member 2 (NR4A2) (Fig. 3), which are known to be involved in various nuclear hormone receptor-signalling pathways with relevance for the human health (Fig. 4).

This RXR-mediated signalling is of general importance for cell differentiation, general development, embryogenesis, cell cycle regulation, apoptosis, general inflammation and immune response, as well as general lipid metabolism as outlined in Figs. 3 and 4 (reviewed in [9, 83, 84]). These summarized physiological pathways can be regulated by RXR-ligand (9CDHRA) – RXR - “plus other NHR” (i.e. LXRs, FXR, PPARs, NR4A2 and VDR)-mediated pathways, but also via RAR - ligand (ATRA) – RAR – RXR – RXR-ligand (9CDHRA) - RXR-regulated pathways and are thereby likely directly dependent on sufficient nutritional vitamin A1 and vitamin A5/X supply.

Many of these physiological pathways rely on complex co-regulatory pathways, involving multiple RXR-LXRs, -PPARs, -NR4A2, -FXR and -VDR-mediated signalling pathways, including RAR - RXR-mediated signalling [11, 12, 85–92]. These parallel signalling pathways require RAR- and RXR-ligands and in consequence the dietary intake of their nutritional precursors. In summary, all these previously mentioned pathways thereby depend on sufficient external nutritional vitamin A1 and vitamin A5/X supply, while nothing is known on further homeostatic pathways to control intracellular concentrations.

**Fig. 5:**
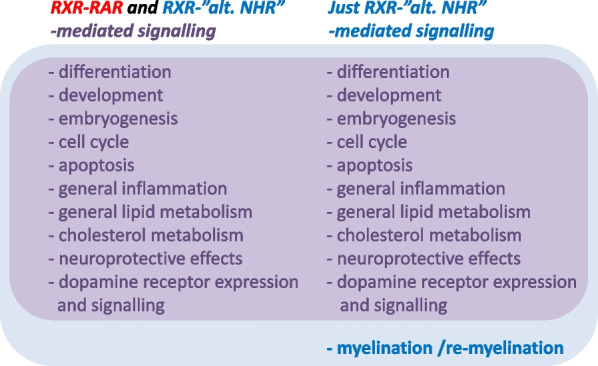
Physiological relevance of summarized overlapping and non-overlapping physiological biochemical pathways mediated via RXR-RAR-mediated signalling and/or RXR- “alternative nuclear hormone receptor (NHR)”-mediated signalling in the mammalian organism

Elucidating vitamin A1 ligand - RAR-mediated signalling-independent pathways is necessary to claim and identify a vitamin A5/X-specific vitamin deficiency. These vitamin A5/X-ligand – RXR —“plus other NHR”- NHR-ligand mediated-signalling pathways may be implicated in insulin/glucose regulation [11, 12, 85–92], cholesterol metabolism [93–95] with a large impact on the cardiovascular system and especially in the nerve system/brain area such as neurogenesis [96], neuroprotection [97, 98], working memory [99, 100], appetite regulation [101], sleep–wake cycle regulation [102], myelination / re-myelination [103, 104] and dopaminergic signalling [105–108] via dopamine 2 receptor (D2R) expressional control and are all mainly PPARs-, FXRs- and LXRs - RXR-mediated pathways. In the majority of all these listed complex physiological regulations a vitamin A1 - RAR-mediated signalling co-occurs, while for myelination / re-myelination [103, 104], this singular mechanisms is clearly non-vitamin A1 - RAR-co-mediated but exclusively dependent on vitamin A5/X - RXR-mediated signalling [103–106]. In consequence, this singular physiological mechanism thereby presents an exclusive vitamin A5/X-dependent physiological mechanism, as displayed in Fig. 5. These summarized vitamin A5/X-dependent physiological mechanisms, in addition to a non-sufficient daily vitamin A5/X intake, may result in a vitamin A5/X-deficiency syndrome, which we will focus in detail on in a follow-up review.

This puts vitamin A5/X in the center as the major switch enabling nuclear hormone receptor-mediated signalling via activation of one side, the RXR-side, of the NHR-heterodimer and thereby enabling a larger array of NHR-mediated signalling pathways, ranging from RARs-, PPARs-, LXRs-, VDR-, FXR- to NR4A2-mediated signalling pathways [7, 8]. The RXR-mediated pathways were excellently summarized by Evans and Mangelsdorf [9], even claiming RXR-mediated signalling starting by RXR-activation, with 9CRA being the “Big Bang” of molecular endocrinology. As summarized in various review articles [24, 40, 67, 83], and based on analytical data, the physiological-relevant existence of 9CRA in the mammalian organism is highly questionable. Therefore, the vitamin A5/X - 9CDHRA - RXR-connection is a new proven theory, putting vitamin A5/X as a nutritional-dependent spark for a real “Big Bang” in human life [9]. In addition to vitamin A5/X - 9CDHRA, also further possible RXR ligands have been described recently [109], such as synthetic analogues including e.g. bexarotene (and related compounds), an anti-cancer drug, could be potentially acting on RXR mediated pathways. Thus, a large array of physiological processes is thereby enabled by vitamin A5/X, summarized in a slightly modified and “corrected” Fig. 4. Physiological processes including cholesterol homeostasis, bile acid homeostasis, fatty acid homeostasis, xenoprotection, basal metabolic rate, calcium- and phosphate-homeostasis and development are vitamin A5/X - RXR-co-regulated or even VA5/X-specific regulated physiological pathways that are important for a large array of crucial life-maintaining functions within the mammalian organism [9].

## Disease specific dysfunctions based on RXR-mediated pathways with a focus on neurological diseases

Retinoid signalling, particularly RXR-mediated pathways, plays a crucial role not only during the development of the central and peripheral nervous system (CNS/PNS), but is also involved in various maintenance functions of the adult CNS. Besides the pivotal involvement of RXR-mediated signalling in the modulation of immune-mediated processes [110–115] and in the cardio-vascular system [9, 10, 83, 116], RXR-mediated signalling has been demonstrated to be critically involved in neuronal homeostasis at numerous levels, as reviewed [117–123]. These various physiological events in the CNS and PNS that depend on RXR-mediated signalling are thus likely dependent on a nutritional supply of vitamin A5/X compounds.

**Fig. 6:**
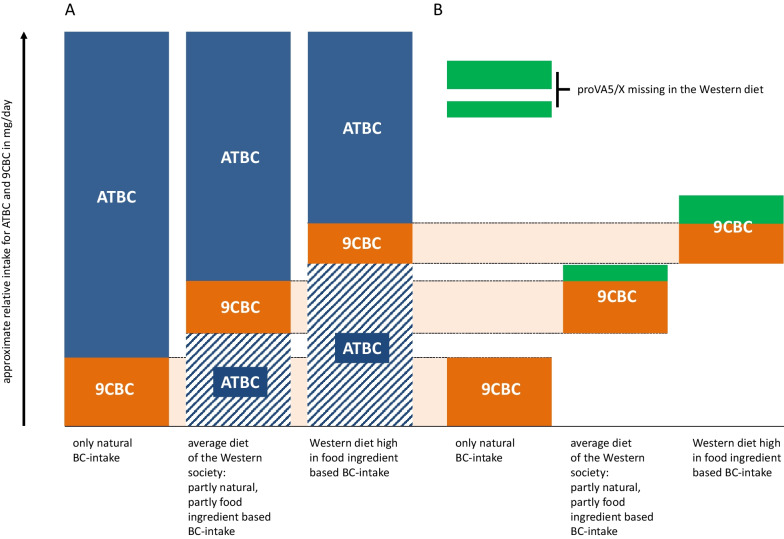
**A** Outlined is the missing micronutrient / vitamin concept based on only natural β-carotene (BC) derived intake of 9-*cis*-β,β-carotene (9CBC) and all-*trans*-β,β-carotene (ATBC), with further increased amounts of food ingredient-based BC-intake present in Western-based diets. **B** Summarized are the missing amounts of 9CBC based on only natural β-carotene (BC) derived intake of 9CBC and ATBC, with further increased amounts of food-ingredient based BC-intake marked with orange bars. Resulting missing 9CBC amounts / provitamin A5 (proVA5/X) in the Western diet are represented by the green bars. For approximate intake recommendations, see Fig. 2

Key processes that are both RXR-mediated and found to be also dysregulated in neurological disorders include cholesterol metabolism [93–95], immune-mediated mechanisms [110–115], myelination / remyelination [103, 104] and dopamine signalling [105–108]. Here, we assume and propose for the first time that a non-sufficient nutritional supply with vitamin A5/X / provitamin A5/X, which are present mainly in fruits and vegetables, as shown in Table 2b and Fig. 6/7, might contribute, in consequence, via their RXR-ligand precursor function, to the comparably large prevalence of neurological disorders in the Western society [124–127].

These neurological disorders represent a growing socioeconomic burden [128, 129] and are expected to become one of the leading causes of disability worldwide along with the projected demographical changes [130, 131]. Current commercial data confirm this importance of neurological diseases, while a large share of all pharma sales in the Western world relies on neuro-pharmaceuticals [132, 133].

Retinoid-, particularly RXR-mediated signalling, has been linked at multiple levels with both, neurodegenerative diseases such as Alzheimer´s disease [98, 117, 122, 134, 135] and Parkinson’s disease [106, 136, 137], inflammation- and myelination-associated disorders, such as the various forms of multiple sclerosis [103, 104] and neurological disorders with a pathophysiological basis in artherosclerosis, including stroke and vascular dementia [117, 122]. Last but not least, there is the group of socio-economically highly relevant psychiatric disorders [121], particularly major depression and schizophrenia [137], which have both been linked to abnormal retinoid signaling [121, 136, 137]. Several of the RXR-mediated mechanisms associated with neurological disorders may be considered “disease-spanning”, including neuroinflammation, synaptic plasticity, dopamine signalling, homeostatic maintenance mechanisms within the CNS, and there are also disease-specific alterations and therapeutic benefits of RXR-signaling.

## Is there a specific vitamin A5/X deficiency?

As analytical monitoring of provitamin A5/X, vitamin A5/X and active derivatives of vitamin A5/X was only recently established for the human body and food items, it is difficult to conclusively associate specific deficiency syndromes with reduced vitamin A5/X levels in the organisms and even more importantly with reduced vitamin A5/X dietary intakes. As important specific functions were already outlined (Figs. 4 and 5), it would appear logic that in these areas potential deficiency symptoms occur, due to low intake of vitamin A5/X and provitamin A5/X.

Especially vegetables, with a focus on leafy and root vegetables (Table 2B), are high in provitamin A5/X content. A reduced intake of vegetables is co-associated with vitamin A5/X - RXR-mediated signalling dependent diseases that entail an increased incidence of neurological diseases [124–127, 138] as well as with cardio-vascular diseases, cancer and immune-disorders/allergies. In consequence, specific physiological mechanisms within the central and peripheral nervous system (as described earlier and in Fig. 5) and a non-sufficient daily vitamin A5/X intake (as outlined earlier and in Fig. 2), may represent a direct link between a low vegetable intake and dysfunctions of the nervous system found in various studies. Especially dietary intake of food items as described earlier (Table 2B) to be rich in 9CBC such as green leafy vegetables, which are usually consumed low in Western-type diets [139], correlate inversely with cognitive decline [140].

Unfortunately, a direct clear connection as indicated by a step-by-step cascade, starting from lower vegetable intake resulting in reduced endogenous vitamin A5/X-derivatives, reduced vitamin A5/X - RXR-mediated signalling and an increased incidence of specific diseases and other RXR-co-associated physiological dysfunctions was thus far not identified. However, summarising all these arguments, a cascade based on scientific evidence starting from food intake towards physiological/patho-physiological functions, is clearly plausible and evident.

## Vitamin A5/X acid and vitamin A5/X / provitamin A5/X as new candidates for food and pharma applications for enabling maintenance and even increased RXR-mediated signalling

Given that all vitamins / provitamins are usually used in food applications to help achieving the recommended daily dietary vitamin intake, this also entails a general suggestion for vitamin A intake. In consequence, this should also be addressed for vitamin A5/X, including provitamin A5/X, and should result in a daily suggested recommendation for their individual intake.

For this reason, the concept of the missing micronutrient has been put forward in this review. We have calculated and proposed recommendations for vitamin A5/X, which are calculated based on natural food sources and provitamin A1 as food ingredients (Fig. 6). For a healthy diet, various national and international health organizations, including the World Health Organization (WHO, [34]), the National Health Service (NHS, [141]) of the United Kingdom and the German Nutrition Society (DGE, [44]) encourage the consumption of at least five portions of fruits and vegetables each day. Unfortunately, based on governmental nutritional surveys, for example in the USA [142], France [143] and Germany [144] a mere 5–25% of our population may reach these daily intake recommendations, resulting in 75–95% of the general population being below the recommended daily dietary suggested intakes. This low daily intake of fruits and vegetables results in postulating that the levels of the newly identified vitamin A5/X / provitamin A5/X, which is mainly present also in fruits and vegetables, are also potentially low in our Western society.

A reduced fruit and vegetable intake results usually in too low intake of vitamin A1 / provitamin A1 and likely also for provitamin A5/X / vitamin A5/X. Unfortunately, only vitamin A1 / provitamin A1 is supplemented to our convenient and readily prepared diet, while the vitamin A5/X / provitamin A5/X is not additionally supplemented. This means that there exists likely a nutritional gap (represented by the green blocks in Fig. 6), a micronutrient gap of provitamin A5/X / vitamin A5/X daily intake, shown and outlined in Fig. 6. A simple supplementation with vitamin A1 / provitamin A1 (shown by the blue dashed line blocks) may not help to maintain related vitamin A5/X / provitamin A5/X levels, as this “normal” provitamin A1 and vitamin A1 are proven to constitute no direct precursors of vitamin A5/X and provitamin A5/X in humans.

In consequence, this suggests that;

- a low additional supplementation of provitamin A5/X / vitamin A5/X is needed when high natural provitamin A(1 + 5) and low supplemental provitamin A(1) is ingested, and
- a high additional supplementation of vitamin A5/X / provitamin A5/X is needed when low natural provitamin A(1 + 5) / high supplemental provitamin A(1) is ingested.

The daily recommended intake amounts were calculated to be in the range of 0.5–1.8 mg of 9CBC per day as a total and 0.2–0.9 mg of 9CBC per day as a currently missing micronutrient amount in the Western-based diets, as calculated in Fig. 2.

Furthermore, 9CDHROL, 9CDHROL-esters, 9CDHRA and 9CDHRA-esters may be used also in pharmaceutical applications with relevance for the CNS/PNS and the cardio-vascular system (CVS) and applications addressing cancer, such as relevant for alternative synthetic RXR-agonists [97, 117, 122, 134].

## We propose a “to do list” for national and international authorities

Due to the fact that a new direct and independent food to ligand to action (physiological relevance) pathway was outlined, national and international authorities should get active in various areas to set up clear governmental regulations and dietary suggestions. We suggest here, summarized in several points, due to the unclear and uncertain general vitamin and especially vitamin A definition and regulation, the following actions:

(A) Define clearly what is vitamin A / provitamin A and define what potential sub-categories such as vitamin A1 / A2 exist and adjust the current nutritional recommendations for DRI and upper limits with relevance for vitamin A, vitamin A1 and vitamin A2.
(B) Define whether vitamin A5/X / provitamin A5/X are fully or partly included in these recommendations, especially considering the double precursor function of 9CBC as provitamin A1 / A5/X.
(C) Define the precursor functions of vitamin A1 / A2 as well as of vitamin A5/X in regard of vitamin A deficiencies, especially under consideration of the RAR- and RXR-ligand precursor concept.
(D) Define gaps and “a to-do list” to clarify main aspects regarding the definition and food regulation aspects for vitamin A1 / A2 and vitamin A5/X.

**Fig. 7:**
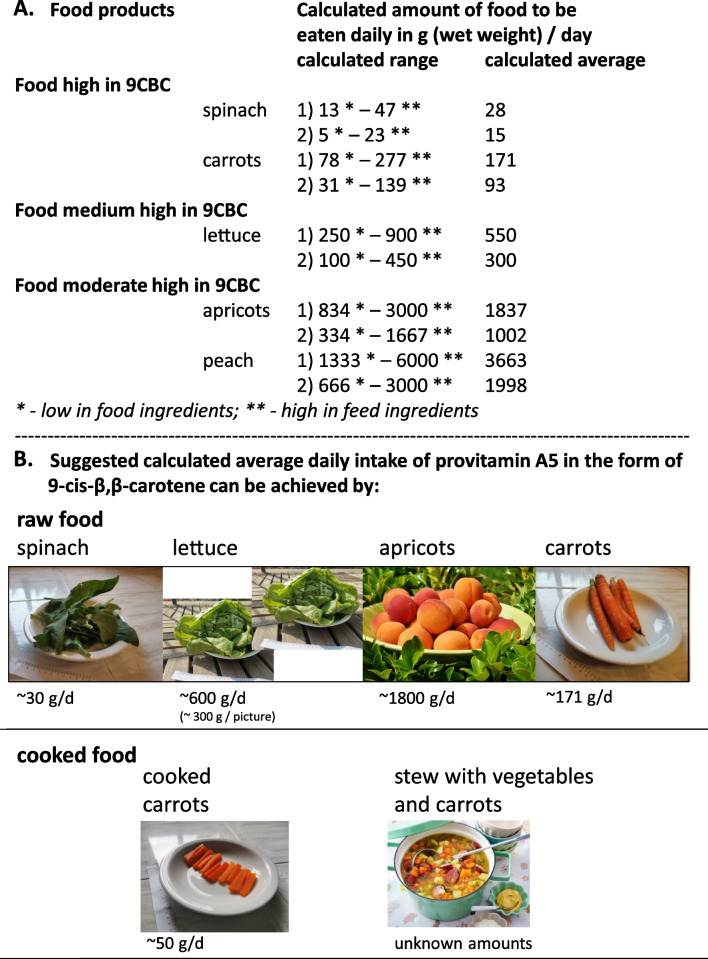
**A** Examples of individual food products needed: (1) To fulfill the total 9-*cis*-β,β-carotene (9CBC) RDI demand of 0.5–1.8 (calculated and further used average of 1.1) mg/day, based on our calculations from Table 1/Fig. 2. (2) Missing 9CBC in the human food chain due to pure all-*trans*-β,β-carotene (ATBC) used in food fortification of 0.2–0.9 (calculated and further used average of 0.6) mg/day, based on calculations from Table 1/Fig. 2. **B** Summary of food items and amounts as examples of a balanced and recommended diet rich in provitamin A5/X in the form of 9CBC based on our current suggested recommendations are based on Table 1/Figs. 2/7A. Selected pictures contain a mm/cm grid for precise size adjustment of the precise food amount described

Based on the summarized data in this review and on existing EFSA-based recommendations regarding BC and vitamin A(1), we propose novel daily recommendations for vitamin A5/X. This would rather constitute a general recommendation for a natural occurring nutrient that is present in natural foods, but could also be included in the diet as a supplement or to a fortified food compound if intakes from natural sources are perceived as low (Table 1, Additional file 1: Table 1, Figs. 2 and 6). This may be especially true due to the exclusive usage of pure ATBC in commonly used food ingredients. Further calculated amounts of natural food products that would cover low, medium or high intakes of provitamin A5/X were additionally proposed based on their content of provitamin A5/X (Fig. 7).

## Conclusions

When novel concepts are identified, which partly overlap with existing knowledge, novel guidelines and clear borders and overlaps must be described and defined. We now suggest vitamin A5/X / provitamin A5/X as a new vitamin A sub-category, termed “vitamin A5”, or even as a novel independent vitamin category, with the suggested name “vitamin X”. In addition, for vitamin A5/X and with a focus on provitamin A5/X, nutritional regulations comparable to DRIs/DRVs applied should be suggested, based on known nutritional and mechanistic pathways for optimal healthy, natural based, as well as further fortification-based recommendations.

### Supplementary Information

**Additional file 1**. Supplementary table 1.

## Data Availability

Data are available on request.

## References

[1] Wald G. The photoreceptor process in vision. In: Handbook physiology. Washington D.C.: American Physiological Society; 1959. p. 671–692.

[2] Wald G (1945). Human vision and the spectrum. Science.

[3] Wald G (1935). Vitamin a in eye tissues. J Gen Physiol.

[4] Petkovich M, Brand NJ, Krust A, Chambon P (1987). A human retinoic acid receptor which belongs to the family of nuclear receptors. Nature.

[5] Giguere V, Ong ES, Segui P, Evans RM (1987). Identification of a receptor for the morphogen retinoic acid. Nature.

[6] Petkovich M (1992). Regulation of gene expression by vitamin A: the role of nuclear retinoic acid receptors. Annu Rev Nutr.

[7] Mangelsdorf DJ, Thummel C, Beato M, Herrlich P, Schutz G, Umesono K, Blumberg B, Kastner P, Mark M, Chambon P (1995). The nuclear receptor superfamily: the second decade. Cell.

[8] Mangelsdorf DJ, Evans RM (1995). The RXR heterodimers and orphan receptors. Cell.

[9] Evans RM, Mangelsdorf DJ (2014). Nuclear receptors, RXR, and the big bang. Cell.

[10] Evans RM (2005). The nuclear receptor superfamily: a rosetta stone for physiology. Mol Endocrinol.

[11] Shulman AI, Mangelsdorf DJ (2005). Retinoid x receptor heterodimers in the metabolic syndrome. N Engl J Med.

[12] Chawla A, Repa JJ, Evans RM, Mangelsdorf DJ (2001). Nuclear receptors and lipid physiology: opening the X-files. Science.

[13] Kastner P, Messaddeq N, Mark M, Wendling O, Grondona JM, Ward S, Ghyselinck N, Chambon P (1997). Vitamin A deficiency and mutations of RXRalpha, RXRbeta and RARalpha lead to early differentiation of embryonic ventricular cardiomyocytes. Development.

[14] Ghyselinck NB, Dupe V, Dierich A, Messaddeq N, Garnier JM, Rochette-Egly C, Chambon P, Mark M (1997). Role of the retinoic acid receptor beta (RARbeta) during mouse development. Int J Dev Biol.

[15] McCarthy P, Cerecedo LR (1952). Vitamin A deficiency in the mouse. J Nutr.

[16] Aykroyd WR (1930). Functional night-blindness due to vitamin A deficiency. Lancet.

[17] Wolfe JM, Salter HP (1930). Vitamin A deficiency in the Albino mouse. J Nutr.

[18] Blackfan KD, Wolbach SB (1933). Vitamin A deficiency in infants. J Pediatr.

[19] Mukherjee S, Date A, Patravale V, Korting HC, Roeder A, Weindl G (2006). Retinoids in the treatment of skin aging: an overview of clinical efficacy and safety. Clin Interv Aging.

[20] Zasada M, Budzisz E (2019). Retinoids: active molecules influencing skin structure formation in cosmetic and dermatological treatments. Postepy Dermatol Alergol.

[21] Beckenbach L, Baron JM, Merk HF, Loffler H, Amann PM (2015). Retinoid treatment of skin diseases. Eur J Dermatol.

[22] Altucci L, Leibowitz MD, Ogilvie KM, de Lera AR, Gronemeyer H (2007). RAR and RXR modulation in cancer and metabolic disease. Nat Rev Drug Discov.

[23] Altucci L, Gronemeyer H (2001). The promise of retinoids to fight against cancer. Nat Rev Cancer.

[24] Bohn T, Desmarchelier C, El SN, Keijer J, van Schothorst EM, Rühl R, Borel P (2019). B-carotene in the human body-metabolic acitivation pathways—from digestion to tissue distribution. Proc Nutr Soc.

[25] Bohn T, Desmarchelier C, Dragsted LO, Nielsen CS, Stahl W, Ruhl R, Keijer J, Borel P (2017). Host-related factors explaining interindividual variability of carotenoid bioavailability and tissue concentrations in humans. Mol Nutr Food Res.

[26] Böhm V, Lietz G, Olmedilla-Alonso B, Phelan D, Reboul E, Banati D, Borel P, Corte-Real J, de Lera AR, Desmarchelier C (2020). From carotenoid intake to carotenoid blood and tissue concentrations-implications for dietary intake recommendations. Nutr Rev.

[27] Rollman O, Vahlquist A (1985). Vitamin A in skin and serum–studies of acne vulgaris, atopic dermatitis, ichthyosis vulgaris and lichen planus. Br J Dermatol.

[28] Escaron AL, Green MH, Tanumihardjo SA (2009). Plasma turnover of 3,4-didehydroretinol (vitamin A2) increases in vitamin A-deficient rats fed low versus high dietary fat. J Lipid Res.

[29] Howell JM, Thompson JN, Pitt GA (1967). Reproduction and vision in rats maintained on a retinol-free diet containing 3-dehydroretinol (vitamin A2). Br J Nutr.

[30] Cama HR, Dalvi PD, Morton RA, Salah MK (1952). Studies in vitamin A. XXI. Retinene2 and vitamin A2. Biochem J.

[31] Shantz EM (1948). Isolation of pure vitamin A2. Science.

[32] Moise AR, Isken A, Dominguez M, de Lera AR, von Lintig J, Palczewski K (2007). Specificity of zebrafish retinol saturase: formation of all-trans-13,14-dihydroretinol and all-trans-7,8- dihydroretinol. Biochemistry.

[33] Babino D, Golczak M, Kiser PD, Wyss A, Palczewski K, von Lintig J. The biochemical basis of vitamin A3 production in athopod vision. ACS Chem Biol. 2016; in press. 10.1021/acschembio.5b00967 PMC4841470 26811964

[34] WHO. Vitamin and mineral requirements in human nutrition; report of a joint FAO/WHO expert consultation. Bankok: WHO/FAO of the UN; 1998.

[35] Krezel W, Rivas A, Szklenar M, Ciancia M, Alvarez R, de Lera AR, Rühl R (2021). Vitamin A5/X, a new food to lipid hormone concept for a nutritional ligand to control RXR-mediated signaling. Nutrients.

[36] Codex-alimentarius. Guidelines on nutrient labelling CAC/GL 2-1985. WHO/FAO. 2017.

[37] EFSA. Scientific opinion on dietary reference values for vitamin A. 13 2015: 4028. 10.2903/j.efsa.2015.4149 PMC13151645 42109809

[38] Krezel W, Rühl R, de Lera AR (2019). Alternative retinoid X receptor (RXR) ligands. Mol Cell Endocrinol.

[39] de Lera AR, Krezel W, Rühl R (2016). An endogenous mammalian retinoid X receptor ligand, at last!. ChemMedChem.

[40] Rühl R, Krezel W, de Lera AR (2018). 9-Cis-13,14-dihydroretinoic acid, a new endogenous mammalian ligand of retinoid X receptor and the active ligand of a potential new vitamin A category: vitamin A5. Nutr Rev.

[41] Rühl R, Krzyzosiak A, Niewiadomska-Cimicka A, Rochel N, Szeles L, Vaz B, Wietrzych-Schindler M, Alvarez S, Szklenar M, Nagy L (2015). 9-cis-13,14-dihydroretinoic acid is an endogenous retinoid acting as RXR ligand in mice. PLoS Genet.

[42] Bohn T, de Lera ÁR, Landrier JF, Carlsen H, Merk D, Todt T, Renaut J, Rühl R. State-of-the-art methodological investigation of carotenoid activity and metabolism—from organic synthesis via metabolism to biological activity—exemplified by a novel retinoid signalling pathway. Food Funct. 2023; in press. 10.1039/d2fo02816f 36562448

[43] Bohn T, de Lera AR, Landrier JF, Rühl R (2022). Carotenoid metabolites, their tissue and blood concentrations in humans and further bioactivity via retinoid receptor-mediated signalling. Nutr Res Rev.

[44] DGE. Vitamin A; Empfohlene Zufuhr. Deutsche Gesellschaft für Ernährung e.V. 2020.

[45] EFSA. Scientific opinion on the re-evaluation of mixed carotenes (E 160a (i)) and beta-carotene (E 160a (ii)) as a food additive. EFSA J. 2012;10:2593. 10.2903/j.efsa.2012.2593 PMC13151842 42112025

[46] EFSA. Statement on the safety of ß-carotene use in heavy smokers. EFSA J. 2012;10:2953.

[47] Omenn GS, Goodman GE, Thornquist MD, Balmes J, Cullen MR, Glass A, Keogh JP, Meyskens FL, Valanis B, Williams JH (1996). Effects of a combination of beta carotene and vitamin A on lung cancer and cardiovascular disease. N Engl J Med.

[48] ATBC-cancer-prevention-group. The effect of vitamin E and beta carotene on the incidence of lung cancer and other cancers in male smokers. N Engl J Med 1994;330:1029–1035. 10.1056/NEJM199404143301501 8127329

[49] Wang XD, Liu C, Bronson RT, Smith DE, Krinsky NI, Russell M (1999). Retinoid signaling and activator protein-1 expression in ferrets given beta-carotene supplements and exposed to tobacco smoke. J Natl Cancer Inst.

[50] Liu C, Wang XD, Bronson RT, Smith DE, Krinsky NI, Russell RM (2000). Effects of physiological versus pharmacological beta-carotene supplementation on cell proliferation and histopathological changes in the lungs of cigarette smoke-exposed ferrets. Carcinogenesis.

[51] Lee LM, Leung CY, Tang WW, Choi HL, Leung YC, McCaffery PJ, Wang CC, Woolf AS, Shum AS (2012). A paradoxical teratogenic mechanism for retinoic acid. Proc Natl Acad Sci U S A.

[52] Homnick DN, Spillers CR, Cox SR, Cox JH, Yelton LA, DeLoof MJ, Oliver LK, Ringer TV (1995). Single- and multiple-dose-response relationships of beta-carotene in cystic fibrosis. J Pediatr.

[53] MayoClinic. Drugs and supplements; beta carotene (Oral route).

[54] Mathews-Roth MM (1984). Treatment of erythropoietic protoporphyria with beta-carotene. Photodermatol.

[55] Micozzi MS, Brown ED, Taylor PR, Wolfe E (1988). Carotenodermia in men with elevated carotenoid intake from foods and beta-carotene supplements. Am J Clin Nutr.

[56] Bresnahan KA, Davis CR, Tanumihardjo SA (2014). Relative vitamin A values of 9-cis- and 13-cis-beta-carotene do not differ when fed at physiological levels during vitamin A depletion in Mongolian gerbils (*Meriones unguiculatus*). Br J Nutr.

[57] Sauberlich HE, Hodges RE, Wallace DL, Kolder H, Canham JE, Hood J, Raica N, Lowry LK (1974). Vitamin A metabolism and requirements in the human studied with the use of labeled retinol. Vitam Horm.

[58] Khan NC, West CE, de Pee S, Bosch D, Phuong HD, Hulshof PJ, Khoi HH, Verhoef H, Hautvast JG (2007). The contribution of plant foods to the vitamin A supply of lactating women in Vietnam: a randomized controlled trial. Am J Clin Nutr.

[59] EU-SCF. Opinion of the Scientific comitee on Food on safety of use of beta carotene from dietary sources. EU-scientific opinions 2000; SCF/CS/ADD/COL/159 Final.

[60] Drummond JC, Coward KH (1920). Researches on the fat-soluble accessory factor (vitamin A). Biochem J.

[61] Wolbach SB, Howe PR (1925). Tissue changes following deprivation of fat-soluble a vitamin. J Exp Med.

[62] Moore T (1929). Vitamin A and carotene: the association of vitamin A activity with carotene in the carrot root. Biochem J.

[63] Heyman RA, Mangelsdorf DJ, Dyck JA, Stein RB, Eichele G, Evans RM, Thaller C (1992). 9-cis retinoic acid is a high affinity ligand for the retinoid X receptor. Cell.

[64] Levin AA, Sturzenbecker LJ, Kazmer S, Bosakowski T, Huselton C, Allenby G, Speck J, Kratzeisen C, Rosenberger M, Lovey A (1992). 9-cis retinoic acid stereoisomer binds and activates the nuclear receptor RXR alpha. Nature.

[65] Allenby G, Bocquel MT, Saunders M, Kazmer S, Speck J, Rosenberger M, Lovey A, Kastner P, Grippo JF, Chambon P (1993). Retinoic acid receptors and retinoid X receptors: interactions with endogenous retinoic acids. Proc Natl Acad Sci U S A.

[66] Shih TW, Shealy YF, Strother DL, Hill DL (1986). Nonenzymatic isomerization of all-trans- and 13-cis-retinoids catalyzed by sulfhydryl groups. Drug Metab Dispos.

[67] Paik J, Vogel S, Piantedosi R, Sykes A, Blaner WS, Swisshelm K (2000). 9-cis-retinoids: biosynthesis of 9-cis-retinoic acid. Biochemistry.

[68] Maeda T, Perusek L, Amengual J, Babino D, Palczewski K, von Lintig J (2011). Dietary 9-cis-beta, beta-carotene fails to rescue vision in mouse models of leber congenital amaurosis. Mol Pharmacol.

[69] Tryggvason K, Romert A, Eriksson U (2001). Biosynthesis of 9-cis-retinoic acid in vivo. The roles of different retinol dehydrogenases and a structure-activity analysis of microsomal retinol dehydrogenases. J Biol Chem.

[70] Wang XD, Krinsky NI, Benotti PN, Russell RM (1994). Biosynthesis of 9-cis-retinoic acid from 9-cis-beta-carotene in human intestinal mucosa in vitro. Arch Biochem Biophys.

[71] Davidi L, Pick U (2017). Novel 9-cis/all-trans beta-carotene isomerases from plastidic oil bodies in *Dunaliella bardawil* catalyze the conversion of all-trans to 9-cis beta-carotene. Plant Cell Rep.

[72] Xu Y, Harvey PJ (2019). Red light control of beta-carotene isomerisation to 9-cis beta-carotene and carotenoid accumulation in *Dunaliella salina*. Antioxidants (Basel).

[73] Lin H, Wang R, Qian Q, Yan M, Meng X, Fu Z, Yan C, Jiang B, Su Z, Li J (2009). DWARF27, an iron-containing protein required for the biosynthesis of strigolactones, regulates rice tiller bud outgrowth. Plant Cell.

[74] Alder A, Jamil M, Marzorati M, Bruno M, Vermathen M, Bigler P, Ghisla S, Bouwmeester H, Beyer P, Al-Babili S (2012). The path from beta-carotene to carlactone, a strigolactone-like plant hormone. Science.

[75] Marx M, Stuparic M, Schieber A, Carle R (2003). Effects of thermal processing on trans-cis-isomerization of ß-carotene in carrot juices and carotene-containing preparations. Food Chem.

[76] Aman R, Schieber A, Carle R (2005). Effects of heating and illumination on trans-cis isomerization and degradation of beta-carotene and lutein in isolated spinach chloroplasts. J Agric Food Chem.

[77] Lessin JW, Catigani GL, Schwartz SJ (1997). Quantification of cis-trans isomers of provitamin A carotenoids in fresh and processed fruits and vegetables. J Agric Food Chem.

[78] EFSA. Safety of the proposed extension of use of synthetic ß-carotene [E 160a(ii)] in foods for special medical purposes in young children. EFSA J. 2016;14. 10.2903/j.efsa.2016.4487 PMC13151579 42109449

[79] Codex-alimentarius. Proposed draft amendments to the international system for food additives (CX 36-1989). WHO/FAO 2019; CX/FA 19/51/12.

[80] EU-comission-regulation. Laying down specifications for food additives listes in annex II and II to regulation No 1333/2008 of the European parliament and of the council. Off J Eur Union. 2012;L83/1.

[81] EU-Comission-regulation. Laying down specifications for food additives listes in annex II and II to regulation No 1333/2008 of the European parliament and of the council. Off J Eur Union. 2020;200/771.

[82] Wells ML, Potin P, Craigie JS, Raven JA, Merchant SS, Helliwell KE, Smith AG, Camire ME, Brawley SH (2017). Algae as nutritional and functional food sources: revisiting our understanding. J Appl Phycol.

[83] Desvergne B (2007). RXR: from partnership to leadership in metabolic regulations. Vitam Horm.

[84] Szanto A, Narkar V, Shen Q, Uray IP, Davies PJ, Nagy L (2004). Retinoid X receptors: X-ploring their (patho)physiological functions. Cell Death Differ.

[85] Yamauchi T, Waki H, Kamon J, Murakami K, Motojima K, Komeda K, Miki H, Kubota N, Terauchi Y, Tsuchida A (2001). Inhibition of RXR and PPARgamma ameliorates diet-induced obesity and type 2 diabetes. J Clin Invest.

[86] Pinaire JA, Reifel-Miller A (2007). Therapeutic potential of retinoid x receptor modulators for the treatment of the metabolic syndrome. PPAR Res.

[87] Leibowitz MD, Ardecky RJ, Boehm MF, Broderick CL, Carfagna MA, Crombie DL, D'Arrigo J, Etgen GJ, Faul MM, Grese TA (2006). Biological characterization of a heterodimer-selective retinoid X receptor modulator: potential benefits for the treatment of type 2 diabetes. Endocrinology.

[88] Mukherjee R, Davies PJ, Crombie DL, Bischoff ED, Cesario RM, Jow L, Hamann LG, Boehm MF, Mondon CE, Nadzan AM (1997). Sensitization of diabetic and obese mice to insulin by retinoid X receptor agonists. Nature.

[89] Singh Ahuja H, Liu S, Crombie DL, Boehm M, Leibowitz MD, Heyman RA, Depre C, Nagy L, Tontonoz P, Davies PJ (2001). Differential effects of rexinoids and thiazolidinediones on metabolic gene expression in diabetic rodents. Mol Pharmacol.

[90] Kane MA, Folias AE, Pingitore A, Perri M, Obrochta KM, Krois CR, Cione E, Ryu JY, Napoli JL (2010). Identification of 9-cis-retinoic acid as a pancreas-specific autacoid that attenuates glucose-stimulated insulin secretion. Proc Natl Acad Sci U S A.

[91] Miyazaki S, Taniguchi H, Moritoh Y, Tashiro F, Yamamoto T, Yamato E, Ikegami H, Ozato K, Miyazaki J (2010). Nuclear hormone retinoid X receptor (RXR) negatively regulates the glucose-stimulated insulin secretion of pancreatic ss-cells. Diabetes.

[92] Liu YL, Sennitt MV, Hislop DC, Crombie DL, Heyman RA, Cawthorne MA (2000). Retinoid X receptor agonists have anti-obesity effects and improve insulin sensitivity in Zucker fa/fa rats. Int J Obes Relat Metab Disord.

[93] Ahuja HS, Szanto A, Nagy L, Davies PJ (2003). The retinoid X receptor and its ligands: versatile regulators of metabolic function, cell differentiation and cell death. J Biol Regul Homeost Agents.

[94] Parikh M, Patel K, Soni S, Gandhi T (2014). Liver X receptor: a cardinal target for atherosclerosis and beyond. J Atheroscler Thromb.

[95] Zolberg Relevy N, Bechor S, Harari A, Ben-Amotz A, Kamari Y, Harats D, Shaish A (2015). The inhibition of macrophage foam cell formation by 9-cis beta-carotene is driven by BCMO1 activity. PLoS ONE.

[96] Simandi Z, Horvath A, Cuaranta-Monroy I, Sauer S, Deleuze JF, Nagy L (2017). RXR heterodimers orchestrate transcriptional control of neurogenesis and cell fate specification. Mol Cell Endocrinol.

[97] Dickey AS, Sanchez DN, Arreola M, Sampat KR, Fan W, Arbez N, Akimov S, Van Kanegan MJ, Ohnishi K, Gilmore-Hall SK (2017). PPARdelta activation by bexarotene promotes neuroprotection by restoring bioenergetic and quality control homeostasis. Sci Transl Med.

[98] Mariani MM, Malm T, Lamb R, Jay TR, Neilson L, Casali B, Medarametla L, Landreth GE (2017). Neuronally-directed effects of RXR activation in a mouse model of Alzheimer's disease. Sci Rep.

[99] Wietrzych M, Meziane H, Sutter A, Ghyselinck N, Chapman PF, Chambon P, Krezel W (2005). Working memory deficits in retinoid X receptor gamma-deficient mice. Learn Mem.

[100] Wietrzych-Schindler M, Szyszka-Niagolov M, Ohta K, Endo Y, Perez E, de Lera AR, Chambon P, Krezel W (2011). Retinoid X receptor gamma is implicated in docosahexaenoic acid modulation of despair behaviors and working memory in mice. Biol Psychiatry.

[101] Ogilvie KM, Saladin R, Nagy TR, Urcan MS, Heyman RA, Leibowitz MD (2004). Activation of the retinoid X receptor suppresses appetite in the rat. Endocrinology.

[102] Murillo-Rodriguez E, Millan-Aldaco D, Arankowsky-Sandoval G, Yamamoto T, Cid L, Monteiro D, Rocha NB, Telles-Correia D, Teixeira DS, Veras AB (2020). The retinoid X receptor: a nuclear receptor that modulates the sleep-wake cycle in rats. Psychopharmacology.

[103] Huang JK, Jarjour AA, Nait Oumesmar B, Kerninon C, Williams A, Krezel W, Kagechika H, Bauer J, Zhao C, Baron-Van Evercooren A (2011). Retinoid X receptor gamma signaling accelerates CNS remyelination. Nat Neurosci.

[104] Meffre D, Shackleford G, Hichor M, Gorgievski V, Tzavara ET, Trousson A, Ghoumari AM, Deboux C, Nait Oumesmar B, Liere P (2014). Liver X receptors alpha and beta promote myelination and remyelination in the cerebellum. Proc Natl Acad Sci U S A.

[105] Krzyzosiak A, Szyszka-Niagolov M, Wietrzych M, Gobaille S, Muramatsu S, Krezel W (2010). Retinoid x receptor gamma control of affective behaviors involves dopaminergic signaling in mice. Neuron.

[106] Friling S, Bergsland M, Kjellander S (2009). Activation of retinoid X receptor increases dopamine cell survival in models for Parkinson's disease. BMC Neurosci.

[107] Samad TA, Krezel W, Chambon P, Borrelli E (1997). Regulation of dopaminergic pathways by retinoids: activation of the D2 receptor promoter by members of the retinoic acid receptor-retinoid X receptor family. Proc Natl Acad Sci U S A.

[108] Krezel W, Ghyselinck N, Samad TA, Dupe V, Kastner P, Borrelli E, Chambon P (1998). Impaired locomotion and dopamine signaling in retinoid receptor mutant mice. Science.

[109] de Almeida NR, Conda-Sheridan M (2019). A review of the molecular design and biological activities of RXR agonists. Med Res Rev.

[110] Stephensen CB, Borowsky AD, Lloyd KC (2007). Disruption of Rxra gene in thymocytes and T lymphocytes modestly alters lymphocyte frequencies, proliferation, survival and T helper type 1/type 2 balance. Immunology.

[111] Szeles L, Poliska S, Nagy G, Szatmari I, Szanto A, Pap A, Lindstedt M, Santegoets SJ, Ruhl R, Dezso B (2010). Research resource: transcriptome profiling of genes regulated by RXR and its permissive and nonpermissive partners in differentiating monocyte-derived dendritic cells. Mol Endocrinol.

[112] Heine G, Hollstein T, Treptow S, Radbruch A, Worm M (2017). 9-cis retinoic acid modulates the type I allergic immune response. J Allergy Clin Immunol.

[113] Treptow S, Grun J, Scholz J, Radbruch A, Heine G, Worm M (2020). 9-cis retinoic acid and 1.25-dihydroxyvitamin D3 drive differentiation into IgA(+) secreting plasmablasts in human naive B cells. Eur J Immunol.

[114] Kalsotra A, Du L, Wang Y, Ladd PA, Kikuta Y, Duvic M, Boyd AS, Keeney DS, Strobel HW (2007). Inflammation resolved by retinoid X receptor-mediated inactivation of leukotriene signaling pathways. Faseb J.

[115] Nunez V, Alameda D, Rico D, Mota R, Gonzalo P, Cedenilla M, Fischer T, Bosca L, Glass CK, Arroyo AG (2010). Retinoid X receptor alpha controls innate inflammatory responses through the up-regulation of chemokine expression. Proc Natl Acad Sci U S A.

[116] Evans RM, Barish GD, Wang YX (2004). PPARs and the complex journey to obesity. Nat Med.

[117] Moutinho M, Codocedo JF, Puntambekar SS, Landreth GE (2018). Nuclear receptors as therapeutic targets for neurodegenerative diseases: lost in translation. Annu Rev Pharmacol Toxicol.

[118] Woloszynowska-Fraser MU, Kouchmeshky A, McCaffery P (2020). Vitamin A and retinoic acid in cognition and cognitive disease. Annu Rev Nutr.

[119] Shearer KD, Stoney PN, Morgan PJ, McCaffery PJ (2012). A vitamin for the brain. Trends Neurosci.

[120] Stoney PN, McCaffery P (2016). A vitamin on the mind: new discoveries on control of the brain by vitamin A. World Rev Nutr Diet.

[121] van Neerven S, Kampmann E, Mey J (2008). RAR/RXR and PPAR/RXR signaling in neurological and psychiatric diseases. Prog Neurobiol.

[122] Moutinho M, Landreth GE (2017). Therapeutic potential of nuclear receptor agonists in Alzheimer's disease. J Lipid Res.

[123] Zetterstrom RH, Lindqvist E, Mata de Urquiza A, Tomac A, Eriksson U, Perlmann T, Olson L (1999). Role of retinoids in the CNS: differential expression of retinoid binding proteins and receptors and evidence for presence of retinoic acid. Eur J Neurosci.

[124] Bishwajit G, O'Leary DP, Ghosh S, Sanni Y, Shangfeng T, Zhanchun F (2017). Association between depression and fruit and vegetable consumption among adults in South Asia. BMC Psychiatry.

[125] Fitzgerald KC, Tyry T, Salter A, Cofield SS, Cutter G, Fox R, Marrie RA (2017). Diet quality is associated with disability and symptom severity in multiple sclerosis. Neurology.

[126] Joseph J, Cole G, Head E, Ingram D (2009). Nutrition, brain aging, and neurodegeneration. J Neurosci.

[127] Spencer SJ, Korosi A, Laye S, Shukitt-Hale B, Barrientos RM (2009). Food for thought: how nutrition impacts cognition and emotion. NPJ Sci Food.

[128] GBD-2016-Neurology-Collaborators. Global, regional, and national burden of neurological disorders, 1990–2016: a systematic analysis for the Global Burden of Disease Study 2016. Lancet Neurol. 2019;18:459–480. 10.1016/S1474-4422(18)30499-X PMC6459001 30879893

[129] EFSA Panel on Dietetic Products N, Allergies (NDA), European Food Safety Authority (EFSA). Scientific opinion on detary reference values for vitamin A. EFSA J. 2015;13:4028. 10.2903/j.efsa.2015.4149 PMC13151645 42109809

[130] Patel V, Saxena S, Lund C, Thornicroft G, Baingana F, Bolton P, Chisholm D, Collins PY, Cooper JL, Eaton J (2018). The Lancet Commission on global mental health and sustainable development. Lancet.

[131] Hyman SE (2008). A glimmer of light for neuropsychiatric disorders. Nature.

[132] Trautmann S, Rehm J, Wittchen HU (2016). The economic costs of mental disorders: do our societies react appropriately to the burden of mental disorders?. EMBO Rep.

[133] Biopharma-Dealmakers. A view into the central nervous system disorders market. Nat Biopharmadeal. 2020; B37–B39.

[134] Pierrot N, Lhommel R, Quenon L, Hanseeuw B, Dricot L, Sindic C, Maloteaux JM, Octavea JN, Ivanoiu A (2016). Targretin improves cognitive and biological markers in a patient with Alzheimer's disease. J Alzheimers Dis.

[135] Sodhi RK, Singh N (2014). Retinoids as potential targets for Alzheimer's disease. Pharmacol Biochem Behav.

[136] Reay WR, Cairns MJ (2019). The role of the retinoids in schizophrenia: genomic and clinical perspectives. Mol Psychiatry.

[137] Tsai SY, Catts VS, Fullerton JM, Corley SM, Fillman SG, Weickert CS (2017). Nuclear receptors and neuroinflammation in schizophrenia. Mol Neuropsychiatry.

[138] Nguyen B, Ding D, Mihrshahi S (2017). Fruit and vegetable consumption and psychological distress: cross-sectional and longitudinal analyses based on a large Australian sample. BMJ Open.

[139] Johnson M, McElhenney WH, Egnin M (2019). Influence of green leafy vegetables in diets with an elevated omega-6: omega-3 fatty acid ratio on rat blood pressure, plasma lipids, antioxidant status and markers of inflammation. Nutrients.

[140] Morris MC, Wang Y, Barnes LL, Bennett DA, Dawson-Hughes B, Booth SL (2017). Nutrients and bioactives in green leafy vegetables and cognitive decline: prospective study. Neurology.

[141] NHS. The eat well guide/https://www.nhs.uk/live-well/eat-well/the-eatwell-guide. 2021.

[142] Casagrande SS, Wang Y, Anderson C, Gary TL (2007). Have Americans increased their fruit and vegetable intake? The trends between 1988 and 2002. Am J Prev Med.

[143] Tavoularis G, Hebel P. Fruits es legumes: le Francais suivent de moins la recommandation. Centre de reseraches pour létude et lóbservation des condition de vie. 2017. p. 292.

[144] Mensink GB, Truthmann J, Rabenberg M, Heidemann C, Haftenberger M, Schienkiewitz A, Richter A (2013). Fruit and vegetable intake in Germany: results of the German health interview and examination survey for adults (DEGS1). Bundesgesundheitsblatt Gesundheitsforschung Gesundheitsschutz.

[145] EFSA. Tolerable upper intake levels for vitamins and minerals. EFSA J. 2006.

[146] Institute-of-Medicine-Food-and-Nutrition-Board. Dietary reference intakes for vitamin A, vitamin K, arsenic, boron, chromium, copper, iodine, iron, manganese, molybdenum, nickel, silicon, vanadium, and zinc. Washington: National Academy Press; 2001. 25057538

[147] Expert-group-on-vitamis-and-minerals. safe upper levels for vitamins and minerals. Food Standards Agency. 2003.

[148] Stahl W, Sundquist AR, Hanusch M, Schwarz W, Sies H (1993). Separation of beta-carotene and lycopene geometrical isomers in biological samples. Clin Chem.

[149] Stahl W, Schwarz W, Sundquist AR, Sies H (1992). cis-trans isomers of lycopene and beta-carotene in human serum and tissues. Arch Biochem Biophys.

[150] Clinton SK, Emenhiser C, Schwartz SJ, Bostwick DG, Williams AW, Moore BJ, Erdman JW (1996). Cis-trans lycopene isomers, carotenoids, and retinol in the human prostate. Cancer Epidemiol Biomark Prev.

[151] Johnson EJ, Qin J, Krinsky NI, Russell RM (1997). Beta-carotene isomers in human serum, breast milk and buccal mucosa cells after continuous oral doses of all-trans and 9-cis beta-carotene. J Nutr.

[152] Ben-Amotz A, Fishler R (1998). Analysis of carotenoids with emphasis on 9-cis-beta-carotene in vegetables and fruits commonly consumed in israel. Food Chem.

[153] Khoo HE, Prasad KN, Kong KW, Jiang Y, Ismail A (2011). Carotenoids and their isomers: color pigments in fruits and vegetables. Molecules.

[154] Berni P, Chitchumroonchokchai C, Canniatti-Brazaca SG, De Moura FF, Failla ML (2015). Comparison of content and in vitro bioaccessibility of provitamin A carotenoids in home cooked and commercially processed orange fleshed sweet potato (*Ipomea batatas* Lam). Plant Foods Hum Nutr.

[155] Murador DC, Mercadante AZ, de Rosso VV (2016). Cooking techniques improve the levels of bioactive compounds and antioxidant activity in kale and red cabbage. Food Chem.

[156] Stahl W, Schwarz W, Sies H (1993). Human serum concentrations of all-trans beta- and alpha-carotene but not 9-cis beta-carotene increase upon ingestion of a natural isomer mixture obtained from *Dunaliella salina* (Betatene). J Nutr.

[157] Shaish A, Harari A, Hananshvili L, Cohen H, Bitzur R, Luvish T, Ulman E, Golan M, Ben-Amotz A, Gavish D (2006). 9-cis beta-carotene-rich powder of the alga *Dunaliella bardawil* increases plasma HDL-cholesterol in fibrate-treated patients. Atherosclerosis.

[158] Rotenstreich Y, Belkin M, Sadetzki S, Chetrit A, Ferman-Attar G, Sher I, Harari A, Shaish A, Harats D (2013). Treatment with 9-cis beta-carotene-rich powder in patients with retinitis pigmentosa: a randomized crossover trial. JAMA Ophthalmol.

[159] Meshi A, Belkin A, Koval T, Kornhouser T, Assia EI, Rotenstreich Y (2015). An experimental treatment of ocular quinine toxicity with high-dose 9-cis beta-carotene. Retin Cases Brief Rep.

