# Supplementary material for: Human nutritional relevance and suggested nutritional guidelines for vitamin A5/X and provitamin A5/X
Source: Nutr Metab (Lond). 2023 Aug 15;20:34. doi: 10.1186/s12986-023-00750-3 (PMC10426203; doi:10.1186/s12986-023-00750-3)
Supplement: Supplementary file 1 — Additional file 1. Supplementary table 1. [file 12986_2023_750_MOESM1_ESM.docx]

**Supplementary table 1**.

Intake recommendations for vitamin A and provitamin A / β-carotene

**A. Selected intake recommendations for vitamin A in vitamin A equivalents (VAE)**

| **Organization** | **National RDI’s or similar for**  **vitamin A**  **(µg RE/d or µg RAE/d for DACH)** | **National UL or similar for**  **vitamin A**  **(µg RE/d)** |
| --- | --- | --- |
| EFSA PRI and UL^1^, men / women, adults | 750 / 650 | 3000 |
| USDA-RDA and UL^2^, adult male / female (>19 y) | 900 / 700 | 3000 |
| WHO-FAO^3^, adult men and women (19 - 65 y), µg RE/d | 600 / 500 | N/A |
| DACH^4^, adult men / women (>19y)  **Range adult man**  **adult woman**  EFSA-PRI and UL^1^, pregnant women | 850 / 750  **600-900**  **500-750**  700 | N/A  **3000**  **3000**  3000 |
| USDA-RDA and UL^2^, adult pregnant women  WHO-FAO^3^, pregnant women  DACH^4^, pregnant women  **Range pregnant woman**  EFSA-PRI and UL^1^, lactating women  USDA-RDA and UL^2^, adult lactating women  WHO-FAO^3^, lactating women  DACH^4^, lactating women  **Range lactating woman**  USDA-RDA and UL^2^, children (0 - 18 y)  WHO-FAO^3^, children (0 - 18 y  DACH^4^, children (0 - 18y)  **Range children**  UK-EVM-GL & SUL^5^ | 770  800  800  **700-800**  1300  1300  850  1300  **850-1300**  300 – 900  400 - 600  300 – 950  **300-950**  1500 | 3000  N/A  N/A  **3000**  3000  3000  N/A  N/A  **3000**  600 – 2800  N/A  N/A  **600-2800**  N/A |

References: ^1^ EFSA: DRV-PRI and UL [145]; ^2^USDA: DRI-RDA and UL [146]; ^3^WHO/FAO: Safe recommended intake [34,45], ^4^DACH: German, Austrian and Swiss Recommended Dietary Intakes, 2020 [44], ^5^ UK-EVM**:** United Kingdom-Expert Committee on Minerals and Vitamins - GL [147].

Abbreviations: ADI: acceptable daily intake; DGE: German Nutrition Society; DRI: dietary reference intakes; DRV: dietary references values; EFSA: European Food Safety Authority; RDA: Recommended dietary allowance; RDI: recommended daily intake; RAE: retinol activity equivalents; RE: retinol equivalents; RUL: recommended upper limit; CRN: Council for Responsible Nutrition Washington, DC; DGE: German Nutrition Society; GL: guidance level; PRI: population reference intake; RDA: recommended dietary allowance; OSL: observed safe level; SUL: safe upper level; UL: tolerable upper intake level; USDA: US Department of Agriculture; WHO/FAO: World Health Organization/Food and Agricultural Organization.
